# Identification of Initial Colonizing Bacteria in Dental Plaques from Young Adults Using Full-Length 16S rRNA Gene Sequencing

**DOI:** 10.1128/mSystems.00360-19

**Published:** 2019-09-03

**Authors:** Yukari Ihara, Toru Takeshita, Shinya Kageyama, Rie Matsumi, Mikari Asakawa, Yukie Shibata, Yuki Sugiura, Kunio Ishikawa, Ichiro Takahashi, Yoshihisa Yamashita

**Affiliations:** a Section of Preventive and Public Health Dentistry, Division of Oral Health, Growth and Development, Faculty of Dental Science, Kyushu University, Fukuoka, Japan; b Section of Orthodontics and Dentofacial Orthopedics, Division of Oral Health, Growth and Development, Faculty of Dental Science, Kyushu University, Fukuoka, Japan; c OBT Research Center, Faculty of Dental Science, Kyushu University, Fukuoka, Japan; d Department of Biomaterials, Faculty of Dental Science, Kyushu University, Fukuoka, Japan; Dalhousie University

**Keywords:** 16S rRNA, PacBio Sequel, dental plaque, initial colonizer, saliva

## Abstract

Development of dental plaque begins with the adhesion of salivary bacteria to the acquired pellicle covering the tooth surface. In this study, we collected *in vivo* dental plaque formed on hydroxyapatite disks for 6 h from 74 young adults and identified initial colonizing taxa based on full-length 16S rRNA gene sequences. A long-read, single-molecule sequencer, PacBio Sequel, provided 100,109 high-quality full-length 16S rRNA gene sequence reads from the early plaque microbiota, which were assigned to 90 oral bacterial taxa. The microbiota obtained from every individual mostly comprised the 21 predominant taxa with the maximum relative abundance of over 10% (95.8 ± 6.2%, mean ± SD), which included *Streptococcus* species as well as nonstreptococcal species. A hierarchical cluster analysis of their relative abundance distribution suggested three major patterns of microbiota compositions: a Streptococcus mitis/*Streptococcus* sp. HMT-423-dominant profile, a Neisseria sicca/Neisseria flava/Neisseria mucosa-dominant profile, and a complex profile with high diversity. No notable variations in the community structures were associated with the dental caries status, although the total bacterial amounts were larger in the subjects with a high number of caries-experienced teeth (≥8) than in those with no or a low number of caries-experienced teeth. Our results revealed the bacterial taxa primarily involved in early plaque formation on hydroxyapatite disks in young adults.

**IMPORTANCE** Selective attachment of salivary bacteria to the tooth surface is an initial and repetitive phase in dental plaque development. We employed full-length 16S rRNA gene sequence analysis with a high taxonomic resolution using a third-generation sequencer, PacBio Sequel, to determine the bacterial composition during early plaque formation in 74 young adults accurately and in detail. The results revealed 21 bacterial taxa primarily involved in early plaque formation on hydroxyapatite disks in young adults, which include several streptococcal species as well as nonstreptococcal species, such as Neisseria sicca/*N*. *flava*/*N*. *mucosa* and Rothia dentocariosa. Given that no notable variations in the microbiota composition were associated with the dental caries status, the maturation process, rather than the specific bacterial species that are the initial colonizers, is likely to play an important role in the development of dysbiotic microbiota associated with dental caries.

## Abstract (precis)

Selective attachment of salivary bacteria to the tooth surface is an initial and repetitive phase in dental plaque development. We employed full-length 16S rRNA gene sequence analysis with a high taxonomic resolution using a third-generation sequencer, PacBio Sequel, to determine the bacterial composition during early plaque formation in 74 young adults accurately and in detail. The results revealed 21 bacterial taxa primarily involved in early plaque formation on hydroxyapatite disks in young adults, which include several streptococcal species as well as nonstreptococcal species, such as Neisseria sicca/*N*. *flava*/*N*. *mucosa* and Rothia dentocariosa. Given that no notable variations in the microbiota composition were associated with the dental caries status, the maturation process, rather than the specific bacterial species that are the initial colonizers, is likely to play an important role in the development of dysbiotic microbiota associated with dental caries.

## INTRODUCTION

Dental plaque is a polymicrobial biofilm formed on the surface of teeth and is a direct etiological factor of dental caries and periodontitis, which are the two major causes of tooth loss. The development of plaque microbiota is a dynamic process that is initiated with an ordered attachment of microorganisms to the tooth surface (1). This long-term accumulation results in an increase in biomass as well as a compositional shift owing to environmental changes within the biofilm (2, 3), which lead to a greater risk of oral diseases.

Selective adhesion of salivary bacteria to a cleaned tooth surface is an initial and repetitive phase in dental plaque development. Multiple studies have identified early-colonizing taxa capable of attaching to the acquired pellicle covering the enamel, which has been accomplished using several different approaches, such as cultivation (4), a checkerboard hybridization technique (5), an oral microbe identification microarray (6), a microarray and a culture collection (7), and an 16S rRNA gene clone library (8). These studies consistently demonstrated that the early plaque microbiota is constituted primarily by *Streptococcus* species, in particular Streptococcus mitis and Streptococcus oralis, with a subset of nonstreptococcal species, such as *Neisseria*, *Rothia*, and *Gemella*. In contrast, Diaz et al. (8) indicated that the microbiota composition varies on a subject-specific basis in terms of bacterial diversity and composition. Considering the small sample size of previous studies (2 to 15 participants), further investigations are still needed to understand the composition of early plaque microbiota, including investigation into the variation among individuals. In addition, there are no reports on the bacterial composition of early dental plaque in an Asian population, although geographical differences in oral microbiota had been studied (9, 10).

Advances in sequencing technology have developed in second-generation sequencers, with shorter read length but a much higher throughput than Sanger sequencing, and have enabled us to obtain comprehensive information on dozens of complex microbiota samples simultaneously. Furthermore, long sequencing reads with an average length of >10 kb have become available through recent innovations in single-molecule sequencing technology by Pacific Biosciences. Although the raw sequencing reads provided by this platform are still error prone, multiple observations of the same base of a single molecule is allowed by template DNA circularized by ligated adaptors, resulting in the acquisition of consensus insert sequences with high read accuracy (11). Several studies have conducted accurate and detailed identification of the complex microbial communities based on the full-length bacterial 16S rRNA gene using this platform with the circular consensus sequence (CCS) technique (12–14). In addition, with the latest model of this platform, Sequel achieved approximately a throughput gain of ∼7×, allowing for a much deeper sequence depth than the previous model (RS II).

In this study, we collected early dental plaque formed on hydroxyapatite (HA) disks over the course of 6 h from 74 Japanese young adults using a resin splint (see Fig. S1 in the supplemental material). Following comparison with the salivary bacterial populations using partial 16S rRNA gene sequencing analysis, the bacterial composition of each participant was accurately identified based on full-length 16S rRNA gene sequence data using Sequel. We additionally investigated the association between the interindividual variation of the early plaque microbiota and the dental caries status.

10.1128/mSystems.00360-19.2 FIG S1 Resin splint used in this study. (A) A circular hydroxyapatite disk was set in each buccal side of a maxillary second premolar and held in place by passing a string of dental floss around the teeth. (B) A hydroxyapatite disk of 9 mm in diameter was pressed on thermoplastic mouth guard sheet material. Download FIG S1, JPG file, 0.9 MB. Copyright © 2019 Ihara et al. 2019 Ihara et al. This content is distributed under the terms of the Creative Commons Attribution 4.0 International license.

## RESULTS

This study investigated the bacterial composition of early dental plaque of 74 participants (38 females and 36 males) aged from 20 to 32 years having at least 24 teeth. The number of teeth with caries experience ranged from 0 to 17, and the average was 4.8 ± 4.6 (mean ± SD). No distinct cavity was present in their teeth, although small active caries lesions were found in the 18 participants with dental caries experience. Early plaque samples formed on hydroxyapatite disks (see Fig. S1 in the supplemental material) over the course of 6 h were collected from each subject, in addition to their saliva samples.

**FIG 1:**
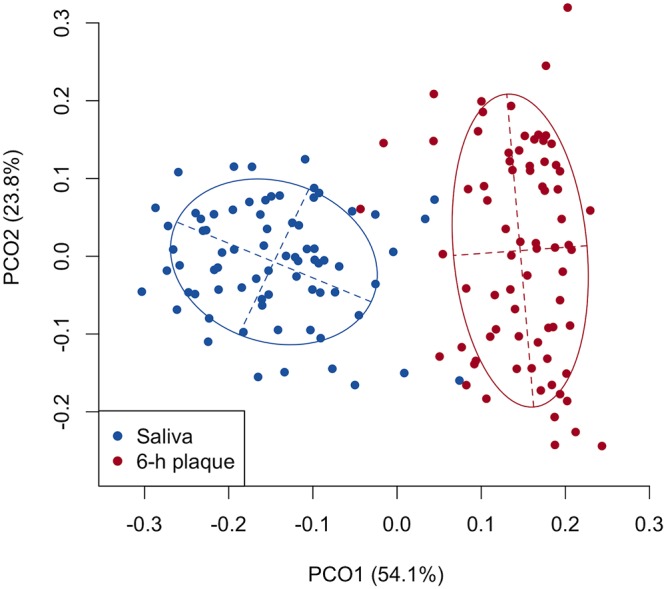
A principal-coordinate analysis (PCoA) plot showing similarity relationships between early plaque and saliva samples from 74 participants using a weighted UniFrac metric. The points corresponding to early plaque (6-h plaque) and saliva are depicted in different colors. The ellipses cover 67% of the samples belonging to each sample type. To correct for the unequal numbers of sequences, we evaluated 5,000 randomly selected sequences per sample.

To confirm that the obtained plaque samples were not simply salivary bacterial populations that were washed onto the disk, the microbiota composition of early plaque samples was compared with that of saliva samples based on the V1-V2 regions of 16S rRNA gene sequence data provided by the second-generation sequencer Ion PGM. A principal-coordinate analysis (PCoA) plot based on the weighted UniFrac metric demonstrated that early plaque microbiota exhibited community structures distinct from those of the salivary bacterial population (Fig. 1). The number of species-level operational taxonomic units (OTUs) identified from each early plaque sample (114.2 ± 25.6, mean ± SD) was significantly lower than that identified from the salivary bacterial populations (156.8 ± 24.7; *P *<* *0.001, Wilcoxon signed-rank test), suggesting that a limited number of bacterial taxa present in saliva attach to the surface of the hydroxyapatite disks. Notable differences between the early plaque and saliva samples were also observed in relative abundances of OTUs commonly (>90% of the participants) detected in the early plaque samples (Table S1). These divergences in the microbiota composition indicated that the early plaque observed in this study was a unique bacterial community formed via selective attachment and growth on the surface of the hydroxyapatite disks.

10.1128/mSystems.00360-19.3 TABLE S1 Candidate bacterial taxa corresponding to the OTUs commonly (>90% of the participants) present in the 6-h plaque microbiota. Download Table S1, DOCX file, 0.01 MB. Copyright © 2019 Ihara et al. 2019 Ihara et al. This content is distributed under the terms of the Creative Commons Attribution 4.0 International license.

**FIG 2:**
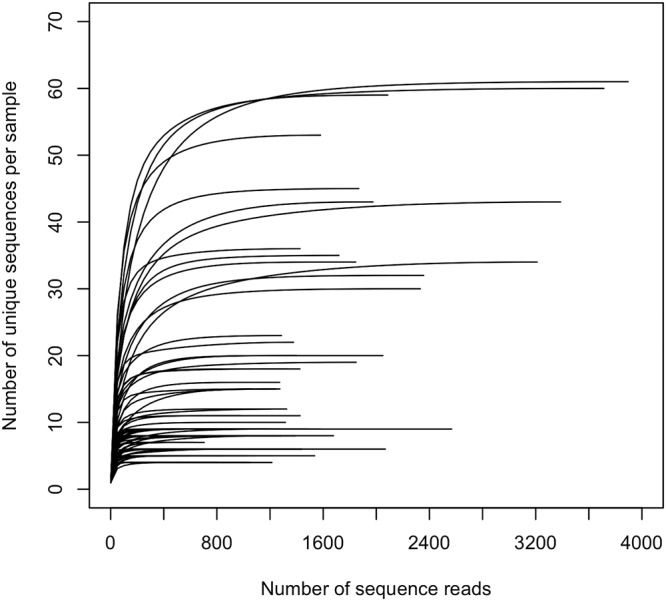
Rarefaction curves for a number of observed unique sequences per sample.

We further applied full-length 16S rRNA gene sequence analysis using a long-read, single-molecule sequencer, PacBio Sequel, to identify the bacterial composition of the early plaque microbiota with higher accuracy. After quality filtering and denoising procedures using R and DADA2 (15), 100,109 CCS reads containing 625 unique full-length 16S rRNA gene sequences were obtained for the 74 early plaque samples (440 to 3,896 reads). The rarefaction curve for the number of unique sequences almost approached a plateau in each sample, although the numbers of sequence reads were not the same among all samples, suggesting that the 16S rRNA gene sequences of initial colonizing bacterial taxa of the 74 young adults were mostly covered by the obtained sequences (Fig. 2). Out of 625 unique sequences, 574 sequences which corresponded to 97,517 reads (97.4% of the total reads) were assigned to 94 oral bacterial species (90 bacterial taxa) deposited in the expanded Human Oral Microbiome Database (eHOMD) (16) with >99% identity (Table S2). However, this study did not distinguish between S. mitis and *Streptococcus* sp. HMT-423; between Streptococcus salivarius and Streptococcus vestibularis; or between Neisseria sicca, Neisseria flava, and Neisseria mucosa, because some of the sequences correspond to both species with the same identity value. The taxonomy of the unassigned 51 sequences which corresponded to the remaining 2,592 reads was determined up to the genus level (Table S2).

10.1128/mSystems.00360-19.4 TABLE S2 Bacterial taxa identified from the early plaque in this study. Download Table S2, DOCX file, 0.02 MB. Copyright © 2019 Ihara et al. 2019 Ihara et al. This content is distributed under the terms of the Creative Commons Attribution 4.0 International license.

**FIG 3:**
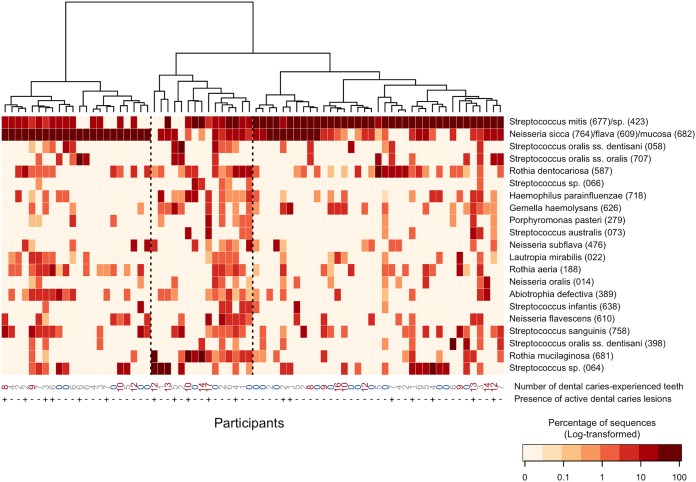
Relative abundances of the 21 predominant taxa in the early plaque of each individual. The percentage of each bacterial taxon (log transformed) is shown in each grid by the color intensity. Both the bacterial taxa and the participants are ordered according to the result of a hierarchical cluster analysis using the Euclidean distance with Ward’s linkage. The clustering results of the bacterial composition are shown as a dendrogram at the top. Oral taxon IDs in eHOMD are given in parentheses following bacterial names. The number of dental caries-experienced teeth of each individual and the presence or absence of active dental caries lesions are described at the bottom of the diagram.

Among the 141 identified taxa, none was shared across the early plaque of all participants. In contrast, the microbiota obtained from each individual mostly comprised the 21 predominant taxa with the maximum relative abundance of over 10% (95.8% ± 6.2%, mean ± SD), implying that a specific subset of oral bacteria constituted the majority of the early plaque microbiota. *Streptococcus* species, as well as nonstreptococcal species, were included in the 21 predominant taxa (Fig. 3), which is consistent with the result of the V1-V2 data (Table S1). S. mitis/*Streptococcus* sp. HMT-423 and *N. sicca/flava*/*mucosa* were the two major taxa observed in the early plaque samples, and the bacterial composition of each individual could be approximately classified into three patterns: an S. mitis/*Streptococcus* sp. HMT-423-dominant profile (right group), an *N. sicca/flava*/*mucosa*-dominant profile (left group), and a complex profile with higher diversity (Fig. 3). Of the other 120 taxa, 106 were present in fewer than five participants, and 79 of them were found in only one participant. However, they occupied only small fractions of the microbiota in each participant (1.8% ± 3.7% and 0.7% ± 1.9%, respectively).

**FIG 4:**
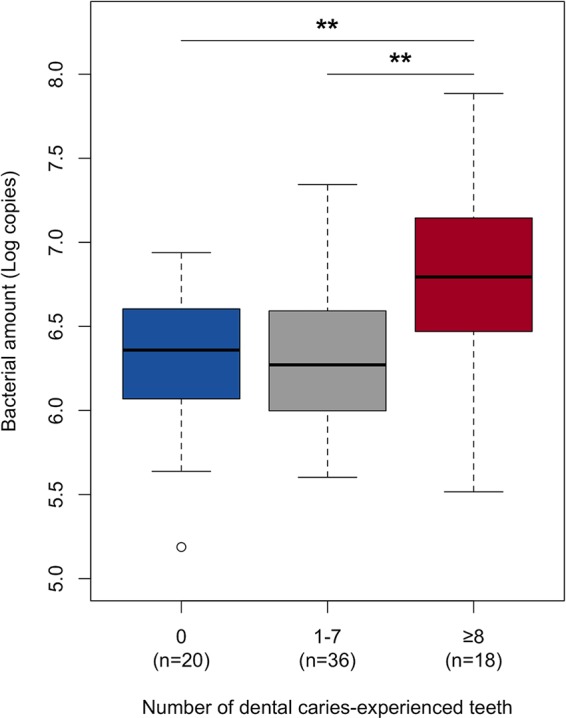
Total bacterial amounts in early plaque microbiota from the subjects with no dental caries-experienced teeth and moderate (1 to 7 teeth) and high numbers (≥8 teeth) of dental caries-experienced teeth. **, *P* < 0.01, Tukey test.

**FIG 5:**
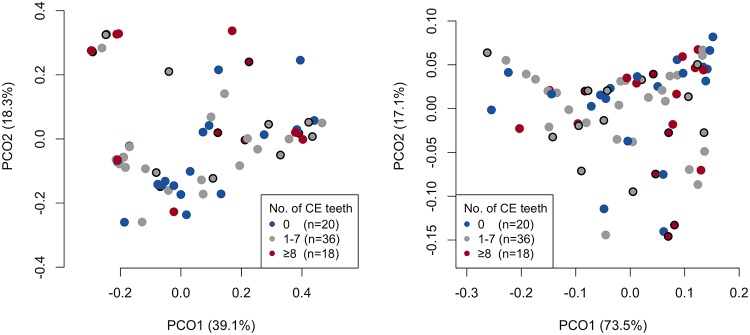
A principal-coordinate analysis (PCoA) plot showing similarity relationships among early plaque samples from 74 subjects using unweighted (left panel) and weighted (right panel) UniFrac metrics. The points corresponding to the subjects with zero dental caries-experienced (CE) teeth or moderate (1 to 7 teeth) and high (≥8 teeth) numbers of CE teeth are depicted in different colors. The points corresponding to the subjects with active dental caries lesions are surrounded by black rings.

To explore interindividual differences of the early plaque microbiota associated with the susceptibility to dental caries, the microbiota profiles were compared among three groups: subjects with no caries-experienced teeth, subjects with a moderate number of caries-experienced teeth (1 to 7), and subjects with a high number of caries-experienced teeth (≥8), which is higher than the average number of caries-experienced teeth in young adults of nearly the same generation in Japanese national survey data (7.4 teeth in adults aged 25 to 35 years [17]). The total bacterial amounts in the early plaque calculated by quantitative PCR analysis were larger in the high-caries group than the no-caries and moderate-caries groups (*P *<* *0.01, Tukey test) (Fig. 4). In contrast, a PCoA plot based on both unweighted and weighted UniFrac metrics implied that notable differences were not observed in the bacterial community structures among the three groups (Fig. 5). No significant difference in dental caries experience status was observed between the three microbiota profiles classified in Fig. 3 (*P* = 0.70, Fisher’s exact test). There was no significant difference in the alpha diversity indices (Table S3), relative abundances (Table S4), and detection frequency (Table S5) of the 21 predominant taxa in the early plaque microbiota among the three groups. No significant difference in the total bacterial amounts in the early plaque was observed between the participants with and without active caries lesions (6.5 ± 0.6 and 6.4 ± 0.4 [log copies], respectively; *P *=* *0.43, Student’s *t* test). The prevalence of the participants with active caries lesions was not significantly different among the three microbiota profiles classified in Fig. 3 (*P* = 0.45, Fisher’s exact test), and both unweighted and weighted UniFrac diagrams also showed that there were no notable differences in the bacterial community structures between the participants with and without the active caries lesions (Fig. 5).

10.1128/mSystems.00360-19.5 TABLE S3 Alpha diversity indices of each individual with a different status of dental caries experience. Download Table S3, DOCX file, 0.01 MB. Copyright © 2019 Ihara et al. 2019 Ihara et al. This content is distributed under the terms of the Creative Commons Attribution 4.0 International license.

10.1128/mSystems.00360-19.6 TABLE S4 Relative abundances of 21 predominant bacterial taxa in 6-h plaque microbiota of subjects with different statuses of dental caries experience. Download Table S4, DOCX file, 0.01 MB. Copyright © 2019 Ihara et al. 2019 Ihara et al. This content is distributed under the terms of the Creative Commons Attribution 4.0 International license.

10.1128/mSystems.00360-19.7 TABLE S5 Detection rate of 21 predominant bacterial taxa in 6-h plaque microbiota of subjects with different statuses of dental caries experience. Download Table S5, DOCX file, 0.01 MB. Copyright © 2019 Ihara et al. 2019 Ihara et al. This content is distributed under the terms of the Creative Commons Attribution 4.0 International license.

## DISCUSSION

This study demonstrates the bacterial composition of early dental plaque formed on hydroxyapatite disks collected from 74 young adults by full-length 16S rRNA gene sequencing using a long-read, single-molecule sequencer, PacBio Sequel. Identification of initial colonizing bacteria using the open-ended molecular approach was conducted in a previous study by Diaz et al. (8). However, the high cost and labor of first-generation Sanger sequencing allowed for the microbiota analysis of only six samples (4-h and 8-h plaque for each of three individuals) with little depth (a total of 531 16S rRNA gene sequences). In this study, we used a third-generation sequencer with the CCS approach and obtained over 100,000 full-length 16S rRNA gene sequences in the early plaques from 74 young adults. The plateaued curve in the rarefaction analysis (Fig. 2) suggests that the sequences obtained almost completely covered the initial colonizing taxa of this study population. Our data add more comprehensive and detailed information on early plaque microbiota with a larger sample size than previously used, which could benefit future investigations of the relationship between early plaque microbiota and dental health status.

The obtained CCS data revealed 21 bacterial taxa occupying over 10% of the individual relative abundance of sequences in any one of the participants (Fig. 3), which could be regarded as important contributors to early plaque formation. The most abundant taxa were *Streptococcus* species, including S. mitis, S. oralis, and Streptococcs sanguinis, which is consistent with previous results (4, 6–8). Our data additionally identified not-yet-named *Streptococcus* species such as *Streptococcus* sp. HMT-064 and *Streptococcus* sp. HMT-066 as dominant initial colonizing microbes. The presence of the former species in the early plaque is in agreement with a previous study (7), whereas that of the latter species is a novel finding to the best of our knowledge. Along with *Streptococcus*, the genera *Neisseria* and *Rothia* occasionally showed the highest relative abundances in the microbiota (23 and two of 74 participants, respectively; data not shown). The majority of *Neisseria* species were *N. sicca*/*flava*/*mucosa*, which were present in the early plaque of all three participants in the study by Diaz et al. (8). The predominant *Neisseria* species in saliva, such as N. flavescens, N. subflava, and N. oralis, were also observed in high proportions in some participants. Of the three identified *Rothia* species, R. dentocariosa and R. mucilaginosa have been reported as initial colonizers (6, 8). Our results add Rothia aeria, which was differentiated from *R. dentocariosa* in 2004 (18), to the principal members of the early plaque microbiota. Gemella haemolysans, Abiotrophia defectiva, and Haemophilus parainfluenzae were additionally found to be members of initial colonizers, as shown in a previous study (8). This study found the dominance of two notable species, including Lautropia mirabilis, which forms the center of cauliflower-like structures composed of multiple species, and Porphyromonas pasteri, which forms a corncob shell within the plaque biofilm, as indicated in the microscopic structural analysis (19). The microbiota of every individual was mostly occupied by these 21 taxa (95.8% ± 6.2%). Based on the relative abundance distribution of these 21 taxa, three compositional patterns are proposed: an S. mitis/*Streptococcus* sp. HMT-423-dominant profile, an *N. sicca/flava*/*mucosa*-dominant profile, and a mixed profile (Fig. 3). In contrast, *Actinomyces* species, which have been indicated as an initial colonizer in earlier studies using cultivation (4) and microscopy (20), were present but were not included owing to their lower relative abundances (maximum relative abundance of *Actinomyces* sp. HMT-175 was 4.2%) compared with those of the above 21 taxa.

The V1-V2 data comparing the early plaque microbiota with the salivary bacterial population show that the primary initial colonizers, such as S. mitis/*Streptococcus* sp. HMT-423, *N. sicca*/*flava*/*mucosa*, *R. aeria*, *G. haemolysans*, and *L. mirabilis*, were present in significantly higher relative abundances in the early plaque microbiota than in saliva (see Table S1 in the supplemental material). Considering that most major members of the salivary bacterial population, including *Prevotella*, *Veillonella*, and *Granulicatella*, were significantly less predominant in the early plaque microbiota (Table S1), these bacteria would be highly capable of attachment and growth on the intact tooth surface. A recent study explored the coaggregation partners of nonstreptococcal taxa, including *R. dentocariosa*, *R. mucilaginosa*, and Haemophilus parainfluenzae, as well as streptococci (7). Our findings support the importance of such investigations and further indicate that the roles of the less well known taxa, such as *L. mirabilis* and *R. aeria,* in the plaque development should be further investigated in future studies.

A previous study using the Sanger sequencing approach provided the 11,368 full-length 16S rRNA gene sequences and reported that several oral bacterial genera were commonly found in the pooled oral samples containing dental plaque from various sites and saliva of all 10 participants, whereas the presence of particular species within that genus was varied among individuals (21). In contrast, even no bacterial genera, including *Streptococcus*, were shared across the early plaque in this study population. These results suggest that selective adhesion to a tooth surface would result in the greater interindividual differences in the bacterial members of the early plaque microbiota than in the bacterial population in the entire oral cavity.

Our previous study demonstrated that 24-h plaque formed on a hydroxyapatite disk from individuals with many caries-experienced teeth contains lower relative abundances of the OTUs corresponding to *N. flava* and *G. haemolysans* than that from caries-free individuals and that the plaque has a different maturation process over 7 days (2). However, although the bacterial composition of the 6-h plaque varied among individuals, as has been indicated previously (8), no notable differences corresponding to the status of dental caries experience was found in this study (Fig. 3 and 5 and Table S3 to Table S5). This result suggests that the membership of the bacterial taxa first attaching to the enamel surface does not play a critical role in the dysbiotic pattern of the plaque microbiota development. In contrast, the total bacterial amount of the 6-h plaque microbiota in the highly caries-experienced group was larger than that in the no-caries and moderate caries-experienced groups (Fig. 4), as observed in 24-h, 48-h, and 72-h plaque formation in a previous study (2), implying that the environmental conditions of intraoral cavities with a higher risk of dental caries would be better for the growth of the initial colonizing bacteria than those with less dental caries risk. Marsh (22) stated that unfavorable environmental conditions additionally drive a compositional shift in the microbiota toward a disease-associated community. The maturation process is therefore more likely to be a more important contributor to succession to the dysbiotic plaque microbiota than the composition of the initial colonizing taxa.

The known dental caries-associated taxa, including mutans streptococci, were not identified in this study (Table S2). They were also not found in the two previous open-ended molecular studies (7, 8) as well as the cultural study (4) using human enamel chips, suggesting that the compositional difference between natural tooth enamel and the hydroxyapatite disk used in this study would not prevent the putative caries pathogens from attachment. On the other hand, one study using a microarray reported that S. mutans and Streptococcus sobrinus were present in the tooth swab samples of some subjects (6). The researchers collected the samples from the coronal two-thirds of buccal dental surfaces of all teeth except the second and third molars, which contain the early plaque formed at the neighboring regions of interproximal area. The discrepancy might be due to such differences in the collection sites. We emphasize that our study mimics the early plaque formed only on the smooth buccal surface of the teeth, but not in the pit and fissure.

The dental plaque develops on the buccal surfaces of the teeth, repeating the contact with the buccal mucosa. However, considering that the positions of the hydroxyapatite disks placed in the oral cavity are closer to the buccal mucosa than are natural tooth surfaces, more buccal epithelial cells and adhering bacteria might contact the disk surface, especially its rim, than the natural tooth surfaces. Nevertheless, the retrieved disks were immersed and rinsed in sterilized phosphate-buffered saline before DNA extraction in order to release the unattached bacteria and cells. We assumed that the bacterial taxa identified in this study could be regarded as the initial colonizing bacteria which attached to the disk surfaces.

We indeed noted one limitation in this study, which is that the subject population was biased toward individuals with relatively good oral conditions. The participants had lower numbers of caries-experienced teeth (4.8 ± 4.6 teeth) compared with a Japanese national survey of nearly the same generation (7.4 teeth [17]), and only two participants had more than 15 caries-experienced teeth (17 teeth at a maximum). Therefore, we might have missed the initial colonizing taxa found only in individuals with severe dental caries experience. Further investigation is still needed to clarify the relationship between the initial colonizing taxa and dental caries susceptibility.

We employed a full-length 16S rRNA gene sequence analysis with a high taxonomic resolution using a third-generation sequencer, PacBio Sequel. This new technique was used to determine the bacterial composition in early plaque samples formed on hydroxyapatite disks from 74 young adults accurately, comprehensively, and in detail. We identified the 21 primary initial colonizing taxa that accounted for most of the microbiota obtained from each individual. This information will be helpful for constructing the polymicrobial biofilm model for early dental plaque and will facilitate future research investigating new approaches in regulating dental biofilms for maintaining good dental health.

## MATERIALS AND METHODS

### Ethics statement.

All participants understood the nature of the study and provided written informed consent. The Ethics Committee of Kyushu University approved the study design as well as the procedure for obtaining informed consent (reference no. 28-127). All experiments were performed in accordance with the approved guidelines.

### Study population, sample collection, and DNA extraction.

Participants were recruited from undergraduate and postgraduate students and the staff at Kyushu University and were aged from 20 to 32 years. Prior to early plaque collection, the dental examination was conducted for each participant by a dentist. Circular hydroxyapatite (HA) disks (9-mm diameter) were fabricated for the collection of early plaque samples because we assumed that careful attention was required for the collection of initial colonizing bacteria without contamination of residual previously formed dental plaque and salivary bacteria. The use of the standardized disks also enabled us to accurately calculate the total amount of attached bacteria for 6 h per unit area. The HA disks were prepared by pressing commercial HA powder (HAP-200; Taihei Co., Osaka, Japan) at 100 MPa onto a uniaxial mold, followed by sintering at 1,200°C for 5 h, and then the disks were polished by SiC sandpaper at #1200 to simulate the surface of tooth enamel. After a meal and tooth brushing, the HA disks pressed on thermoplastic mouth guard sheet material (Erkoflex, 1.0 mm thick; Erkodent, Pfalzgrafenweiler, Germany) were set in each buccal side of the maxillary second premolar and held in place by passing a string of dental floss around the tooth (see Fig. S1 in the supplemental material). The disks were retrieved from the oral cavity 6 h later and placed in a microcentrifuge tube after rinsing with phosphate-buffered saline. Gum-stimulated saliva was also collected from each participant. DNA was extracted from early plaque formed on the disks and saliva using lysozyme and achromopeptidase, as described previously (23). For one subject, DNA extracted from the early plaque sample was inappropriate for later analysis due to its low quality, and therefore, the subject was excluded from the study.

### Ion Torrent 16S rRNA gene sequencing analysis.

Sequencing of the V1-V2 region of 16S rRNA genes of all 148 DNA samples (early plaque and saliva microbiota samples of the 74 subjects) was performed using Ion PGM (Thermo Fisher Scientific, Waltham, MA) with the following primers: 8F (5′-AGA GTT TGA TYM TGG CTC AG 3′) and 338R (5′-TGC TGC CTC CCG TAG GAG T-3′). The detailed procedures of the sequencing and the data processing are described in Text S1 in the supplemental material.

10.1128/mSystems.00360-19.1 TEXT S1 Ion Torrent 16S rRNA gene sequencing analysis. Download Text S1, DOCX file, 0.03 MB. Copyright © 2019 Ihara et al. 2019 Ihara et al. This content is distributed under the terms of the Creative Commons Attribution 4.0 International license.

### Full-length 16S rRNA gene sequencing analysis using PacBio Sequel.

The V1 to V9 regions of 16S rRNA genes from each sample were amplified using the following primers: 8F (5′-AGA GTT TGA TYM TGG CTC AG 3′), with Ion Torrent adaptor A and the sample-specific 8-base tag sequence, and 1492R (5′-GGY TAC CTT GTT ACG ACT T-3′). PCR amplification was carried out using KOD DNA polymerase (Toyobo, Osaka, Japan) under the following cycling conditions: 98°C for 2 min, followed by 30 cycles of 98°C for 15 s, 60°C for 20 s, and 74°C for 90 s. The amplicons were purified using an Agencourt AMPure XP kit (Beckman Coulter, Brea, CA) according to the manufacturer’s instructions. The DNA concentrations and qualities were assessed using a NanoDrop spectrophotometer (NanoDrop Technologies, Wilmington, DE). Equal amounts of DNA were pooled, and the pooled DNA was gel purified using a Wizard SV gel and PCR cleanup system (Promega, Madison, WI). The SMRTbell adaptors were ligated onto the purified amplicons, and the library was sequenced using the Sequel Sequencing kit 2.1 (Pacific Biosciences, Menlo Park, CA) on a long-read, single-molecule, real-time sequencer, PacBio Sequel (Pacific Biosciences). The obtained reads were processed using SMRT Link software version 5.1.0.26412 (Pacific Biosciences), which provided circular consensus sequence (CCS) reads with high accuracy from the raw long sequence reads containing multiple reads of 16S rRNA gene sequence.

The CCS reads were excluded from the analysis if they were ≤1,000 bases, if they were ≥1,700 bases, if they had an average quality score of ≤40, or if they did not include the correct forward and reverse primer sequences, and the remaining CCS reads were assigned to the appropriate sample by examining tag sequences using R. The quality-checked CCS reads were further processed by using the DADA2 pipeline (15) with default settings for PacBio reads (version 1.9.1), which provided the table of exact amplicon sequence variants present in each sample by the denoising and chimera-filtering procedures. The denoised CCS reads were aligned using PyNAST (24), and the UniFrac distance metric (25) was used to determine the dissimilarity between pairs of bacterial communities in QIIME (26). The taxonomy of each denoised CCS sequence was determined using BLAST against 16S rRNA gene sequences of 671 oral bacterial 16S rRNA gene sequences (“Oral” was included in the “Body Site” status) in the expanded Human Oral Microbiome Database (eHOMD 16S rRNA RefSeq version 15.1) (16), and the nearest neighbor taxons with ≥99% identity were selected as candidates for each sequence. The numbers of the denoised CCSs corresponding to the same taxa were combined, and the relative abundances and detection frequencies of each taxon were calculated in R. Some taxa deposited in the eHOMD database are difficult to distinguish even with the use of the full-length 16S rRNA gene sequences because of high sequence similarity among them. For example, only two nucleotides (0.1% of the total 1,497 nucleotides) differ between the sequences of the two distinct *Neisseria* species, *N. sicca* and *N. flava.* When a single denoised CCS corresponded to multiple species with exactly the same identity (which also exceeded 99%), we considered them indistinguishable taxa, and the number of their corresponding sequences was combined. The taxonomy of sequences with no BLAST hit was further determined to the genus level using the RDP classifier with a minimum threshold of 80%.

### Quantitative PCR analysis.

Quantitative PCR analysis of total bacterial amounts in the early plaque sample was performed using the primers 806F (5′-TTA GAT ACC CYG GTA GTC C-3′) and 926R (5′-CCG TCA ATT YCT TTG AGT TT-3′) (27) using a QuantiFast SYBR green PCR kit (Qiagen, Hilden, Germany) according to the manufacturer’s instructions. The 16S rRNA gene of Porphyromonas pasteri was inserted into the vector plasmid pBluescript SKII(+) (Stratagene, La Jolla, CA) and used as a real-time control.

### Statistical analysis.

All statistical analyses were conducted using R version 3.5.1. The similarity relationship assessed using the UniFrac metric is presented in a PCoA plot, which was drawn using R. The Wilcoxon signed-rank test was used to evaluate the difference in the microbiota status among early plaque and saliva samples. Tukey’s test was used to assess the differences in total bacterial amounts of early plaque with different statuses of dental caries experience. Student’s *t* test was used to assess the differences in total bacterial amounts of early plaque with presence or absence of active caries lesions. Kruskal-Wallis analysis was used to assess the differences in microbiota status (alpha diversity indices and relative abundance of each taxon) of early plaque between the subjects with different status of dental caries experience. Fisher’s exact test was conducted to assess the relationship between microbiota status (microbiota patterns and detection rate of each taxon) and different dental caries status.

### Data availability.

The sequence data obtained in this study were deposited in the DDBJ Sequence Read Archive under accession no. DRA008726 to DRA008728.
