# Supplementary figures and images for: Identification of Initial Colonizing Bacteria in Dental Plaques from Young Adults Using Full-Length 16S rRNA Gene Sequencing
Source: mSystems. 2019 Sep 3;4(5):e00360-19. doi: 10.1128/mSystems.00360-19 (PMC6722423; doi:10.1128/mSystems.00360-19)

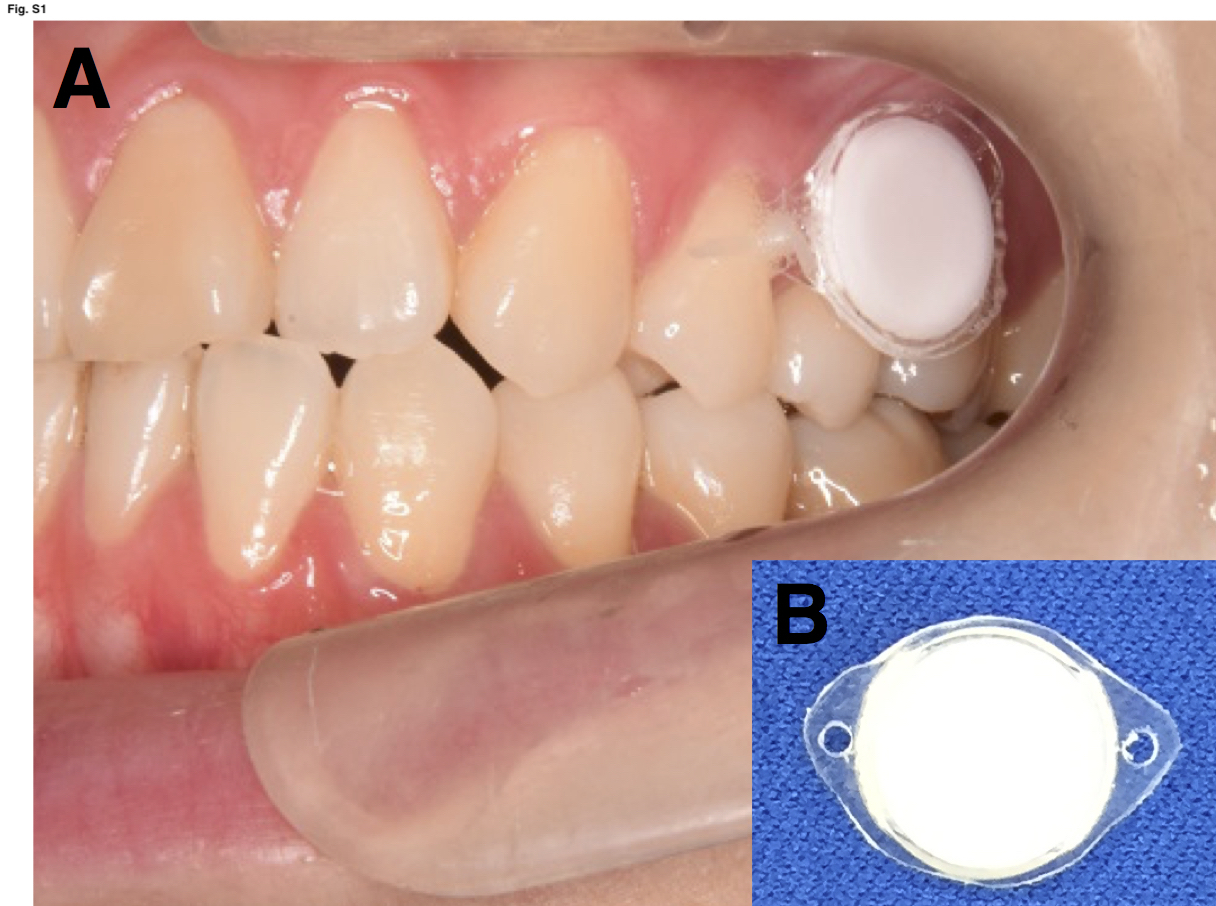

Supplement: FIG S1 [file mSystems.00360-19-sf001.jpg]
