# Supplementary material for: Identification of Initial Colonizing Bacteria in Dental Plaques from Young Adults Using Full-Length 16S rRNA Gene Sequencing
Source: mSystems. 2019 Sep 3;4(5):e00360-19. doi: 10.1128/mSystems.00360-19 (PMC6722423; doi:10.1128/mSystems.00360-19)
Supplement: TABLE S1 [file mSystems.00360-19-st001.docx]

Table S1. Candidate bacterial taxa corresponding to the OTUs commonly (>90% of the participants) present in the 6-h plaque microbiota.

OTU Detection Relative abundances in each microbiota (%)

No. Bacterial candidates corresponding to the OTU rate (%) 6-h plaque Saliva

OTUs corresponding to genus *Streptococcus*

OTU3 *Str. mitis* (677) / sp. (423) / sp. (064) 100 **27.0±15.7***** 9.3±5.0

OTU14 *Str. sanguinis* (758) / *oralis* subsp. *tigurinus* (071) 100 **3.1±3.1***** 0.4±0.3

OTU31 *Str. gordonii* (622) 91.9 **1.1±1.6*****  0.2±0.3

OTU23 *Str. oralis* subsp. *oralis* (707) 98.6 2.8±4.7 1.9±1.4

OTU35 *Str. cristatus* (578) 98.6 1.3±2.1 1.2±1.3

OTU18 Genus *Streptococcus* 100 1.2±2.4 1.1±1.4

OTU172 *Str.* sp. (064) 95.9 0.3±0.6 **0.1±0.2*****

OTU486 Genus *Streptococcus* 91.9 0.2±0.3 **0.9±0.6*****

OTU13 *Str. salivarius* (755) / *vestibularis* (021) 94.6 0.9±1.6 **2.5±2.6*****

OTU24 *Str. australis* (073) 100 1.5±2.3 **2.9±2.1*****

OTUs corresponding to genus *Neisseria*

OTU2 *Nei. sicca* (764) / *mucosa* (682) / *flava* (609) 95.9 **15.0±14.4***** 1.5±2.4

OTU8 *Nei. subflava* (476) / *flavescens* (610) 100 3.1±3.3 **8.6±5.0*****

OTUs corresponding to genus *Rothia*

OTU1 *Rot. mucilaginosa* (681) / *dentocariosa* (587) 100 7.2±8.6 6.3±5.3

OTU4 *Rothia aeria* (188) 97.3 2.6±3.0 **0.4±0.3*****

OTUs corresponding to genus *Haemophilus*

OTU16 *Hae. haemolyticus* (851) / sp. (908) / sp. (036) 97.3 **1.7±2.3*****  0.7±0.7

OTU6 *Hae. parainfluenzae* (718) 100 6.1±6.7 6.2±3.2

OTUs corresponding to other genera

OTU5 *Lautropia mirabilis* (022) 95.9 **2.7±3.6***** 0.3±0.2

OTU29 *Gemella haemolysans* (626) / *morbillorum* (046) 95.9 **2.5±3.3**** 1.1±1.0

OTU48 *Bergeyella* sp. (322) 91.9 0.1±0.1 **0.2±0.1*****

OTU11 *Porphyromonas pasteri* (279) / *catoniae* (283) 91.9 1.5±3.1 **4.9±3.9*****

OTU7 *Fusobacterium periodonticum* (201) / *hwasookii* (953) 94.6 0.7±0.9 **4.2±2.8*****

/ *nucleatum* subsp. *polymorphum* (202)

OTU15 *Veillonella rogosae* (158) / *parvula* (161) / *atypica* (524) 98.6 0.8±1.1 **4.7±2.4*****

/ *dispar* (160) / *denticariosi* (887)

OTU10 *Prevotella melaninogenica* (469) / sp. (314) / sp. (396) 94.6 0.2±0.3 **6.4±4.6*****

/ *scopos* (885) / sp. (313)

OTU9 *Granulicatella adiacens* (534) 100 1.3±0.9 **4.4±2.0*****

Taxon ID in the expanded eHOMD database was given in parentheses following bacterial names. ****P* < 0.001, ***P* < 0.01, **P* < 0.05, Wilcoxon signed rank test adjusted by FDR correction. The values that are significantly higher in the 6h-plaque or in the saliva are shown in bold.
