# Supplementary material for: Identification of Initial Colonizing Bacteria in Dental Plaques from Young Adults Using Full-Length 16S rRNA Gene Sequencing
Source: mSystems. 2019 Sep 3;4(5):e00360-19. doi: 10.1128/mSystems.00360-19 (PMC6722423; doi:10.1128/mSystems.00360-19)
Supplement: TABLE S2 [file mSystems.00360-19-st002.docx]

Table S2. Bacterial taxa identified from the early plaque in this study.

Detection Relative abundance (%)

Bacterial taxa rate (no, (%)) Mean Max

*Abiotrophia defectiva* (389) 29 (39.2) 1.33±2.99 19.6

*Actinomyces dentalis* (888) 1 (1.4) 0±0.02 0.19

*Actinomyces* sp. HMT-169 (169) 5 (6.8) 0.03±0.16 1.23

*Actinomyces* sp. HMT-170 (170) 1 (1.4) 0±0.03 0.24

*Actinomyces* sp. HMT-175 (175) 8 (10.8) 0.16±0.63 4.22

*Actinomyces* sp. HMT-180 (180) 1 (1.4) 0±0.04 0.32

*Aggregatibacter aphrophilus* (545) 1 (1.4) 0±0.01 0.13

*Aggregatibacter* sp. HMT-458 (458) 1 (1.4) 0.01±0.05 0.39

*Aggregatibacter* sp. HMT-512 (512) 1 (1.4) 0±0.03 0.25

Genus *Alloprevotella* dOTU447 1 (1.4) 0.01±0.08 0.67

Genus *Alloprevotella* dOTU559 1 (1.4) 0±0.04 0.32

Genus *Alloprevotella* dOTU616 1 (1.4) 0±0.03 0.23

*Alloprevotella* sp. HMT-308 (308) 1 (1.4) 0.01±0.05 0.41

*Alloprevotella* sp. HMT-473 (473) 6 (8.1) 0.17±0.77 5.71

*Alloprevotella tannerae* (466) 1 (1.4) 0±0.01 0.13

*Bergeyella* sp. HMT-322 (322) 3 (4.1) 0.01±0.03 0.25

Genus *Capnocytophaga* dOTU617 1 (1.4) 0±0.01 0.10

*Capnocytophaga granulosa* (325) 1 (1.4) 0±0.04 0.34

*Cardiobacterium hominis* (633) 1 (1.4) 0±0.01 0.06

Genus *Cloacibacterium* dOTU141 1 (1.4) 0.11±0.94 8.09

Genus *Cloacibacterium* dOTU289 4 (5.4) 0.02±0.13 1.08

Genus *Cloacibacterium* dOTU591 1 (1.4) 0±0.02 0.19

Genus *Cloacibacterium* dOTU65 3 (4.1) 0.14±1.13 9.71

*Corynebacterium durum* (595) 1 (1.4) 0±0.02 0.15

*Dialister invisus* (118) 1 (1.4) 0.01±0.04 0.38

Genus *Escherichia/Shigella* dOTU595 1 (1.4) 0±0.01 0.09

Family *Flavobacteriaceae* dOTU577 1 (1.4) 0±0.02 0.2

Family *Flavobacteriaceae* dOTU581 1 (1.4) 0±0.03 0.22

*Fusobacterium nucleatum* subsp. *animalis* (420) 1 (1.4) 0.01±0.1 0.85

*Fusobacterium periodonticum* (201) 4 (5.4) 0.02±0.11 0.7

*Gemella haemolysans* (626) 27 (36.5) 1.17±2.84 18.72

*Gemella morbillorum* (046) 4 (5.4) 0.02±0.08 0.5

*Gemella sanguinis* (757) 4 (5.4) 0.02±0.16 1.29

*Granulicatella adiacens* (534) 12 (16.2) 0.29±0.79 3.53

*Granulicatella elegans* (596) 6 (8.1) 0.08±0.33 2.44

Genus *Haemophilus* dOTU178 2 (2.7) 0.06±0.43 3.69

Genus *Haemophilus* dOTU325 1 (1.4) 0.01±0.1 0.89

Genus *Haemophilus* dOTU434 2 (2.7) 0.01±0.05 0.31

Genus *Haemophilus* dOTU519 1 (1.4) 0.01±0.05 0.41

Genus *Haemophilus* dOTU562 1 (1.4) 0±0.03 0.25

Genus *Haemophilus* dOTU566 1 (1.4) 0±0.02 0.21

Genus *Haemophilus* dOTU574 1 (1.4) 0±0.03 0.25

Genus *Haemophilus* dOTU582 1 (1.4) 0±0.03 0.22

Genus *Haemophilus* dOTU598 1 (1.4) 0±0.01 0.08

Genus *Haemophilus* dOTU611 1 (1.4) 0±0.02 0.15

Genus *Haemophilus* dOTU613 1 (1.4) 0±0.01 0.13

Genus *Haemophilus* dOTU622 1 (1.4) 0±0.02 0.17

*Haemophilus haemolyticus* (851) 1 (1.4) 0.01±0.05 0.41

*Haemophilus parainfluenzae* (718) 29 (39.2) 1.35±3.29 17.47

*Haemophilus paraphrohaemolyticus* (035) 1 (1.4) 0.01±0.04 0.38

*Haemophilus* sp. HMT-036 (036) 2 (2.7) 0.02±0.13 1.14

*Haemophilus* sp. HMT-908 (908) 3 (4.1) 0.02±0.1 0.65

*Kingella denitrificans* (582) 1 (1.4) 0±0.04 0.34

*Kingella oralis* (706) 1 (1.4) 0±0.01 0.11

*Lautropia mirabilis* (022) 22 (29.7) 0.83±2.41 16.52

*Leptotrichia* sp. HMT-215 (215) 1 (1.4) 0±0.02 0.14

*Neisseria sicca* (764)/*flava* (609)/*mucosa* (682) 62 (83.8) 27.37±26.83 96.72

*Neisseria cinerea* (956) 6 (8.1) 0.07±0.27 1.52

*Neisseria elongata* (598) 3 (4.1) 0.01±0.07 0.54

*Neisseria flavescens* (610) 21 (28.4) 1.47±3.03 16.14

*Neisseria oralis* (014) 16 (21.6) 0.44±1.62 12.6

*Neisseria subflava* (476) 21 (28.4) 1.33±3.89 21.62

*Oribacterium sinus* (457) 1 (1.4) 0±0.02 0.19

Genus *Ottowia* dOTU576 1 (1.4) 0±0.02 0.2

Genus *Porphyromonas* dOTU432 1 (1.4) 0.01±0.1 0.87

Genus *Porphyromonas* dOTU492 1 (1.4) 0.01±0.06 0.51

Genus *Porphyromonas* dOTU514 1 (1.4) 0.01±0.05 0.47

Genus *Porphyromonas* dOTU572 1 (1.4) 0±0.02 0.19

Genus *Porphyromonas* dOTU575 1 (1.4) 0±0.01 0.12

*Porphyromonas pasteri* (279) 14 (18.9) 0.66±2.63 19.21

*Porphyromonas* sp. HMT-278 (278) 1 (1.4) 0±0.03 0.3

*Prevotella aurantiaca* (943) 1 (1.4) 0±0.03 0.24

*Prevotella denticola* (291) 2 (2.7) 0.02±0.12 1.07

Genus *Prevotella* dOTU426 1 (1.4) 0.02±0.17 1.47

Genus *Prevotella* dOTU546 1 (1.4) 0.01±0.07 0.56

*Prevotella intermedia* (643) 1 (1.4) 0.01±0.07 0.63

*Prevotella maculosa* (289) 1 (1.4) 0±0.01 0.13

*Prevotella melaninogenica* (469) 5 (6.8) 0.05±0.2 1.25

*Prevotella nanceiensis* (299) 2 (2.7) 0±0.03 0.19

*Prevotella nigrescens* (693) 1 (1.4) 0±0.01 0.13

*Prevotella oralis* (705) 1 (1.4) 0.01±0.05 0.46

*Prevotella pallens* (714) 1 (1.4) 0±0.01 0.06

*Prevotella pleuritidis* (303) 1 (1.4) 0±0.02 0.16

*Prevotella* sp. HMT-300 (300) 1 (1.4) 0±0.01 0.08

*Prevotella* sp. HMT-301 (301) 1 (1.4) 0±0.02 0.15

*Prevotella* sp. HMT-313 (313) 2 (2.7) 0±0.03 0.29

*Prevotella* sp. HMT-472 (472) 1 (1.4) 0±0.02 0.15

Genus *Pseudomonas* dOTU163 5 (6.8) 0.05±0.18 1.06

Genus *Pseudomonas* dOTU173 4 (5.4) 0.04±0.16 0.83

Genus *Pseudomonas* dOTU254 4 (5.4) 0.03±0.12 0.58

Genus *Pseudomonas* dOTU39 14 (18.9) 0.28±0.72 3.69

Genus *Pseudomonas* dOTU430 2 (2.7) 0.01±0.03 0.22

Genus *Pseudomonas* dOTU457 1 (1.4) 0.01±0.04 0.38

Genus *Pseudomonas* dOTU72 8 (10.8) 0.16±0.5 2.25

Genus *Pseudomonas* dOTU81 7 (9.5) 0.12±0.43 2.30

*Rothia aeria* (188) 32 (43.2) 1.38±2.66 15.93

*Rothia dentocariosa* (587) 43 (58.1) 3.93±8.04 38.62

*Rothia mucilaginosa* (681) 25 (33.8) 2.69±8.21 47.89

*Saccharibacteria* bacterium HMT-346 (346) 1 (1.4) 0±0.03 0.28

*Saccharibacteria* bacterium HMT-352 (352) 1 (1.4) 0±0.01 0.12

*Saccharibacteria* bacterium HMT-356 (356) 2 (2.7) 0.01±0.08 0.61

Genus *Saccharibacteria* dOTU530 1 (1.4) 0±0.04 0.35

*Stomatobaculum longum* (419) 1 (1.4) 0±0.01 0.05

*Stomatobaculum* sp. HMT-097 (097) 1 (1.4) 0±0.01 0.1

*Streptococcus mitis* (677)/sp. (423) 63 (85.1) 36.25±25.76 90.64

*Streptococcus salivarius* (755)/*vestibularis* (021) 13 (17.6) 0.3±0.93 6.03

*Streptococcus australis* (073) 9 (12.2) 0.9±3.87 26.67

*Streptococcus cristatus* (578) 9 (12.2) 0.11±0.36 2.03

Genus *Streptococcus* dOTU106 3 (4.1) 0.12±0.65 4.72

Genus *Streptococcus* dOTU229 1 (1.4) 0.05±0.42 3.61

Genus *Streptococcus* dOTU262 1 (1.4) 0.03±0.26 2.23

Genus *Streptococcus* dOTU336 1 (1.4) 0.02±0.21 1.84

Genus *Streptococcus* dOTU406 1 (1.4) 0.02±0.13 1.14

Genus *Streptococcus* dOTU410 1 (1.4) 0.02±0.19 1.65

Genus *Streptococcus* dOTU476 1 (1.4) 0.01±0.06 0.53

Genus *Streptococcus* dOTU531 1 (1.4) 0±0.03 0.22

Genus *Streptococcus* dOTU538 1 (1.4) 0±0.04 0.32

*Streptococcus gordonii* (622) 14 (18.9) 0.5±1.55 8.84

*Streptococcus infantis* (431) 1 (1.4) 0.04±0.32 2.73

*Streptococcus infantis* (638) 9 (12.2) 0.77±3.09 21.37

*Streptococcus oralis* subsp. *dentisani* (058) 17 (23) 1.94±5.97 32.47

*Streptococcus oralis* subsp. *dentisani* (398) 14 (18.9) 1.04±4.41 34.34

*Streptococcus oralis* subsp. *oralis* (707) 16 (21.6) 2.98±7.63 33.71

*Streptococcus oralis* subsp. *tigurinus* (071) 4 (5.4) 0.21±1.27 9.63

*Streptococcus parasanguinis* (411) 2 (2.7) 0.06±0.51 4.36

*Streptococcus parasanguinis* (721) 1 (1.4) 0.1±0.89 7.64

*Streptococcus sanguinis* (758) 33 (44.6) 2.36±3.83 17.04

*Streptococcus* sp. HMT-057 (057) 1 (1.4) 0.01±0.05 0.43

*Streptococcus* sp. HMT-061 (061) 4 (5.4) 0.11±0.64 5.25

*Streptococcus* sp. HMT-064 (064) 27 (36.5) 5.25±11.59 59.16

*Streptococcus* sp. HMT-066 (066) 7 (9.5) 0.37±1.94 15.7

*Streptococcus* sp. HMT-074 (074) 2 (2.7) 0.08±0.62 5.37

*Tannerella* sp. HMT-286 (286) 1 (1.4) 0±0.02 0.19

*Tannerella* sp. HMT-808 (808) 1 (1.4) 0.01±0.07 0.56

Genus *Tessaracoccus* dOTU542 2 (2.7) 0±0.02 0.14

*Veillonella atypica* (524) 1 (1.4) 0±0.01 0.12

*Veillonella dispar* (160) 2 (2.7) 0.01±0.04 0.29

Genus *Veillonella* dOTU619 1 (1.4) 0±0.01 0.06

*Veillonella parvula* (161) 4 (5.4) 0.05±0.27 2.12

*Veillonella rogosae* (158) 3 (4.1) 0.07±0.41 3.14

*Veillonella* sp. HMT-780 (780) 1 (1.4) 0±0.03 0.29

Taxon ID in eHOMD was given in parentheses following bacterial names. The taxonomy up to the genus level and the serial number of each sequence was described for each sequence with no BLAST hit.
