# Supplementary material for: Identification of Initial Colonizing Bacteria in Dental Plaques from Young Adults Using Full-Length 16S rRNA Gene Sequencing
Source: mSystems. 2019 Sep 3;4(5):e00360-19. doi: 10.1128/mSystems.00360-19 (PMC6722423; doi:10.1128/mSystems.00360-19)
Supplement: TABLE S3 [file mSystems.00360-19-st003.docx]

Table S3. Alpha diversity indices of each individuals with different status of dental caries experience.

Number of dental caries experienced teeth

0 1–7 8–17

(n= 20) (n=36) (n=18) *P*-value

Number of unique sequences 14.8±13.1 15.4±13.5 12.1±11.5 0.402

Shannon diversity index 1.8±0.7 1.8±0.8 1.7±0.6 0.548

Phylogenetic diversity 1±0.5 1±0.5 0.9±0.5 0.658

Kruskal-Wallis analysis was used to assess the differences in alpha diversity indices between the individuals with different status of dental caries experience.
