# Supplementary material for: Identification of Initial Colonizing Bacteria in Dental Plaques from Young Adults Using Full-Length 16S rRNA Gene Sequencing
Source: mSystems. 2019 Sep 3;4(5):e00360-19. doi: 10.1128/mSystems.00360-19 (PMC6722423; doi:10.1128/mSystems.00360-19)
Supplement: TABLE S4 [file mSystems.00360-19-st004.docx]

Table S4. Relative abundances of 21 predominant bacterial taxa in 6-h plaque microbiota of subjects with different status of dental caries experience.

Number of dental caries experienced teeth

0 1–7 8–17

(n= 20) (n=36) (n=18) P-value

*Streptococcus mitis* (677)/sp. (423) 44.5±29.5 29.2±21.4 41.1±26.9 0.449

*Neisseria sicca* (764)/*flava* (609) 24.8±27 33.0±28.0 18.9±22.7 0.449

/*mucosa* (682)

*Streptococcus* sp. (064) 4.1±7.4 5.5±12.0 6.0±14.7 0.856

*Rothia dentocariosa* (587) 3.9±7.2 5.3±9.9 1.2±2.2 0.449

*Streptococcus oralis* ss. *oralis* (707) 0.9±2.4 4.7±9.8 1.7±5.6 0.449

*Rothia mucilaginosa* (681) 1.5±3.9 0.6±1.5 8.1±15.0 0.449

*Streptococcus sanguinis* (758) 2.0±3.4 2.3±3.2 3.0±5.3 0.856

*Streptococcus oralis* ss. *dentisani* (058) 2.3±5.6 2.5±7.4 0.3±1.2 0.512

*Neisseria flavescens* (610) 1.1±2.6 1.6±3.3 1.6±3.1 0.856

*Rothia aeria* (188) 1.1±1.6 1.8±3.1 0.9±2.6 0.449

*Haemophilus parainfluenzae* (718) 1.4±3.4 1.1±3.2 1.8±3.5 0.856

*Neisseria subflava* (476) 2.7±5.7 0.4±1 1.8±4.7 0.662

*Abiotrophia defectiva* (389) 0.8±2.5 1.5±2.1 1.6±4.6 0.449

*Gemella haemolysans* (626) 0.5±1.5 1.3±3.6 1.6±2.3 0.512

*Streptococcus* *oralis* ss. *dentisani* (398) 1±3.5 1.4±5.7 0.3±0.9 0.664

*Streptococcus* *australis* (073) 0.1±0.3 0.3±1 3.1±7.5 0.512

*Lautropia* *mirabilis* (022) 0.2±0.5 0.7±1.4 1.8±4.4 0.664

*Streptococcus* *infantis* (638) 1.1±3.3 1.0±3.7 0±0 0.449

*Porphyromonas* *pasteri* (279) 0.3±0.9 0.4±1.6 1.5±4.8 0.856

*Neisseria* *oralis* (014) 0.3±0.9 0.4±0.8 0.7±3 0.664

*Streptococcus* sp. (066) 0.9±3.5 0.1±0.4 0.3±1.3 0.856

Taxon ID in the expanded Human Oral Microbiome Database was given in parentheses following bacterial names. *P*-values were calculated using Kruskal-Wallis analysis adjusted by FDR correction.
