# Supplementary material for: Identification of Initial Colonizing Bacteria in Dental Plaques from Young Adults Using Full-Length 16S rRNA Gene Sequencing
Source: mSystems. 2019 Sep 3;4(5):e00360-19. doi: 10.1128/mSystems.00360-19 (PMC6722423; doi:10.1128/mSystems.00360-19)
Supplement: TABLE S5 [file mSystems.00360-19-st005.docx]

Table S5. Detection rate of 21 predominant bacterial taxa in 6-h plaque microbiota of subjects with different status of dental caries experience.

Number of dental caries experienced teeth

0 1–7 8–17

(n= 20) (n=36) (n=18) *P*-value

*Streptococcus mitis* (677)/sp. (423) 17(85) 29(80.6) 17(94.4) 0.72

*Neisseria sicca* (764)/*flava* (609) 15(75) 32(88.9) 15(83.3) 0.72

/*mucosa* (682)

*Streptococcus* sp. (064) 9(45) 13(36.1) 5(27.8) 0.736

*Rothia dentocariosa* (587) 13(65) 23(63.9) 7(38.9) 0.72

*Streptococcus oralis* ss. *oralis* (707) 3(15) 11(30.6) 2(11.1) 0.72

*Rothia mucilaginosa* (681) 8(40) 9(25) 8(44.4) 0.72

*Streptococcus sanguinis* (758) 8(40) 18(50) 7(38.9) 0.833

*Streptococcus oralis* ss. *dentisani* (058) 4(20) 11(30.6) 2(11.1) 0.72

*Neisseria flavescens* (610) 4(20) 12(33.3) 5(27.8) 0.736

*Rothia aeria* (188) 10(50) 19(52.8) 3(16.7) 0.567

*Haemophilus parainfluenzae* (718) 9(45) 13(36.1) 7(38.9) 0.867

*Neisseria subflava* (476) 6(30) 8(22.2) 7(38.9) 0.72

*Abiotrophia defectiva* (389) 6(30) 18(50) 5(27.8) 0.72

*Gemella haemolysans* (626) 6(30) 12(33.3) 9(50) 0.72

*Streptococcus oralis* ss. *dentisani* (398) 3(15) 9(25) 2(11.1) 0.72

*Streptococcus australis* (073) 2(10) 3(8.3) 4(22.2) 0.72

*Lautropia mirabilis* (022) 4(20) 12(33.3) 6(33.3) 0.736

*Streptococcus infantis* (638) 4(20) 5(13.9) 0(0) 0.72

*Porphyromonas pasteri* (279) 3(15) 7(19.4) 4(22.2) 0.928

*Neisseria oralis* (014) 5(25) 9(25) 2(11.1) 0.72

*Streptococcus* sp. (066) 2(10) 4(11.1) 1(5.6) 0.928

Taxon ID in the expanded Human Oral Microbiome Database was given in parentheses following bacterial names. *P*-values were calculated using Fisher’s exact test adjusted by FDR correction.
