# Supplementary material for: Identification of Initial Colonizing Bacteria in Dental Plaques from Young Adults Using Full-Length 16S rRNA Gene Sequencing
Source: mSystems. 2019 Sep 3;4(5):e00360-19. doi: 10.1128/mSystems.00360-19 (PMC6722423; doi:10.1128/mSystems.00360-19)
Supplement: TEXT S1 [file mSystems.00360-19-s0001.docx]

Supplemental Methods

**Ion Torrent 16S rRNA gene sequencing analysis.**

The V1–V2 region of 16S rRNA genes from all 148 DNA samples (early plaque and saliva microbiota samples of the 74 subjects) was amplified using the following primers: 8F (5'- AGA GTT TGA TYM TGG CTC AG 3'), with Ion Torrent adaptor A and the sample-specific 8-base tag sequence, and 338R (5'- TGC TGC CTC CCG TAG GAG T -3') with the Ion Torrent trP1 adaptor sequence. PCR amplification, purification, and quantification of each PCR adaptor were performed as previously described (1). Emulsion PCR and enrichment of template-positive particles were performed using an Ion PGM Hi-Q View OT2 kit (Thermo Fisher Scientific, Waltham, MA) with the Ion One Touch 2 system (Thermo Fisher Scientific) and sequencing was performed with the Ion PGM (Thermo Fisher Scientific) using an Ion PGM Hi-Q View sequencing kit (Thermo Fisher Scientific).

The raw sequence reads were quality filtered using a script written in R (2) as described previously (3). The quality-checked reads were assigned to the appropriate samples by examining the tag sequences. Following dereplication, similar sequences were clustered into operational taxonomic units (OTUs) using the “usearch” command in UPARSE (4), with a minimum pairwise identity of 97%, and all quality-checked reads were mapped to each representative OTU using the “usearch_global” command in UPARSE. The representative sequences were aligned using PyNAST (5) in QIIME (6) using a minimum identity of 75% and unaligned reads were excluded from the representative set. Chimeras were additionally removed from the representative sets after being identified using Chimera Slayer (7). The UniFrac metric (8) calculated in QIIME (6) was used to determine the dissimilarities between any pairs of bacterial communities. The taxonomy of each representative sequences was determined using BLAST against 671 oral bacterial 16S rRNA gene sequences ("Oral" was included in the "Body Site" status) in the expanded Human Oral Microbiome Database (eHOMD 16S rRNA RefSeq version 15.1) (9). All species with 98% identity to the representative sequence were selected as candidates for each OTU. The taxonomies of sequences without hits were further determined using the RDP classifier with a minimum support threshold of 80%. Alpha diversity indices and the relative abundances of each OTU were calculated following rarefaction with a depth of 5,000 reads per sample using R.

**REFERENCES**

1. **Takeshita T, Kageyama S, Furuta M, Tsuboi H, Takeuchi K, Shibata Y, Shimazaki Y, Akifusa S, Ninomiya T, Kiyohara Y, Yamashita Y.** 2016. Bacterial diversity in saliva and oral health-related conditions: the Hisayama Study. Sci Rep **6:**22164.

2. **R Core Team.** 2018. R: A language and environment for statistical computing. R Foundation for Statistical Computing, Vienna, Austria. URL <https://www.R-project.org/>.

3. **Asakawa M, Takeshita T, Furuta M, Kageyama S, Takeuchi K, Hata J, Ninomiya T, Yamashita Y.** 2018. Tongue Microbiota and Oral Health Status in Community-Dwelling Elderly Adults. mSphere **3**.

4. **Edgar RC.** 2013. UPARSE: highly accurate OTU sequences from microbial amplicon reads. Nat Methods **10:**996-998.

5. **Caporaso JG, Bittinger K, Bushman FD, DeSantis TZ, Andersen GL, Knight R.** 2010. PyNAST: a flexible tool for aligning sequences to a template alignment. Bioinformatics **26:**266-267.

6. **Caporaso JG, Kuczynski J, Stombaugh J, Bittinger K, Bushman FD, Costello EK, Fierer N, Pena AG, Goodrich JK, Gordon JI, Huttley GA, Kelley ST, Knights D, Koenig JE, Ley RE, Lozupone CA, McDonald D, Muegge BD, Pirrung M, Reeder J, Sevinsky JR, Turnbaugh PJ, Walters WA, Widmann J, Yatsunenko T, Zaneveld J, Knight R.** 2010. QIIME allows analysis of high-throughput community sequencing data. Nat Methods **7:**335-336.

7. **Haas BJ, Gevers D, Earl AM, Feldgarden M, Ward DV, Giannoukos G, Ciulla D, Tabbaa D, Highlander SK, Sodergren E, Methe B, DeSantis TZ, Petrosino JF, Knight R, Birren BW.** 2011. Chimeric 16S rRNA sequence formation and detection in Sanger and 454-pyrosequenced PCR amplicons. Genome Res **21:**494-504.

8. **Lozupone C, Knight R.** 2005. UniFrac: a new phylogenetic method for comparing microbial communities. Appl Environ Microbiol **71:**8228-8235.

9. **Escapa IF, Chen T, Huang Y, Gajare P, Dewhirst FE, Lemon KP.** 2018. New Insights into Human Nostril Microbiome from the Expanded Human Oral Microbiome Database (eHOMD): a Resource for the Microbiome of the Human Aerodigestive Tract. mSystems **3**.
